# Students’ descriptions about experiences beneficial to mental health - a thematic analysis

**DOI:** 10.1186/s12889-025-21846-w

**Published:** 2025-02-19

**Authors:** Anne Skoglund, Sarah Hotham, Agneta Schröder, Øyfrid Larsen Moen

**Affiliations:** 1 Norwegian University of Science and Technology, Gjøvik, 2802 Norway; 2 Centre for Health Services Studies, University of Kent, Cornwallis Building University of Kent, Canterbury, Kent, CT2 7NF UK; 3 University Health Care Research Center, Faculty of Medicine and Health, Örebro University, Örebro, Sweden

**Keywords:** Mental health, Mental health promotion, Students, Universities

## Abstract

University years are an important transitional time for young adults. Recently, an increasing number of students have reported mental health problems. The increasing numbers are an international phenomenon. Qualitative research on mental health promotion for students is, however, scarce. The aim of this study is to explore students’ descriptions of experiences in their student life that are beneficial to mental health. A Norwegian project named “In my experience” collected descriptions, through the web-based tool Sensemaker, from students about experiences that have had an impact on their student life. This study explores the descriptions of experiences beneficial to students’ mental health that the students categorized as having had a positive or very positive impact on their student life. A total of 171 descriptions from students aged 18–29 were analyzed using thematic analysis. Two main themes were identified: becoming a student, which consisted of descriptions about the feeling of a new life as a student, and being a student, which described experiences with managing student life that were beneficial for mental health. Experiences such as being welcomed, being included, belonging to a social group, finding one’s own identity, maturing, and developing were all highlighted in the descriptions. Student societies and other forms of civic engagement and being accepted and included in an academic community were fundamental. A limitation of the study was the relatively low number of male participants, and further research on male students’ descriptions about beneficial experiences is needed.

## Background

Higher education students form a substantial part of society. In Norway, there are almost 300 000 students, approximately 5% of the population. Since 2010, the proportion of students’ self-reporting a mental health problem has increased [1]. According to the Norwegian Students health and wellbeing survey, 42% of the students reported having mental health problems. 13% of the students reported moderate problems, 18% reported serious problems, and 11% reported having both serious and numerous mental problems. The most common problems are depression (11.1%) and anxiety (10.1%). Students are at particularly high risk for developing mental health problems, which can lead to mental health illness. Mental health illness manifests at a young age, and the university years are a major transition and a stressful period of life [2]. An increase in the mental health problems of students is also an international phenomenon. For example in the UK, there has been a sixfold increase in student mental ill health since 2010 [3].

A mentally healthy student life is of great importance for several reasons. Student life forms the basis for the transition to adult life and students’ future roles in community, workplace and family life [4]. It also decreases the chances of dropping out and affects students’ academic performance [5]. Mental health problems are here-and-now problems that may greatly affect students’ wider quality of life.

Investigating positive mental health is relevant for all student, regardless of their mental health status. Fusar- Poli [6] describes mental health as a state of well- being that allows individuals to cope with the normal stresses of life and function productively. A focus on mental health, as outlined in positive psychology, involves emphasizing students’ resources and strengths, as well as efforts to strengthen the communities where students live their daily lives to promote mental well-being (ref kobau 2020).

A Norwegian review found 2,968 studies from the Nordic countries, Greenland and Faroe Islands, on the subject of mental health problems among students [7]. Although there is a body of research on mental health promotion in student life, most of this research is intervention studies and quantitative studies [8]. Therefore, a qualitative, health promoting approach to the problem is necessary [9].

By investigating experiences with a positive impact on the life of students, there is important knowledge to be gained through the students’ own voices about the type and quality of experiences that may contribute to a broader understanding of mental health promotion in student life. Also, knowledge about the contribution to a good student life, and a better foundation for promoting mental health among students may be increased. It may also empower students to make choices in their student life that promotes mental health, empowerment being a key element in health promotion [10, 11]. Also other health promoting approaches such as flourishing [12], salutogenesis [13] and positive psychology may be relevant approaches to promoting mental health in student years. Such knowledge may contribute to the research field about how to improve the way student life is organized by both students themselves, universities, and other stakeholders.

The aim of this study is to describe students’ experiences of student life that are beneficial to mental health. Our research question is:

“How do students experience various aspects of student life to contribute to their mental health?”

## Method

This study employed an descriptive qualitative inductive research design using Braun and Clarkes [14] thematic analysis to describe students’ written experiences. Thematic analysis is a relevant method [15] as the data was approached inductively and analysed as openly as we could.

### Study setting

*In My Experience* is a project that aims to gather knowledge about what is beneficial for students’ mental health. The project was carried out by the student welfare organization in the Norwegian university city of Trondheim [16]. Students documented their experiences using a web- based data collection tool called Sensemaker. The founders of Sensemaker describe it as a way of collecting a description-based repository of insights [17]. The authors of this study were invited by the *In my experience* project management to analyse the data that was collected by Sensemaker to explore the descriptions about positive experiences in students’ lives and their contribution to promoting mental health.

### Participants

Participants were students who fulfilled the inclusion criteria; (1) moved from another region to attend university, as relocation will contribute to greater changes in life situation than if one stays in the same environment. (2) aged between 18 and 29 years and (3) studying for 1–5 years and were both graduate and postgraduate students and (4) their written descriptions were categorized as positive, very positive, neutral or both positive and negative by the participants. Out of 171 participants, 149 were female, 21 were male, and one did not wish to answer questions about gender. Other characteristics such as faculty or specialization was not specified in the filtered data material. The age gap of the participants was as shown in Table 1.

**Table 1:** Age of participants

Age	Number of participants
18–21	38
22–25	111
26–29	22

### Data collection

The basis for the analysis is 171 written descriptions. Data were collected through the web-based data collection tool Sensemaker from November 2020 to October 2021. Sensemaker allows students to write an experience that has had an impact on their student life but also allows the participants to interpret and categorize their own experiences regarding feeling and motivation.

Students were recruited through posters at the campus, posts on social media and by recommendations from friends and students who were employed by the *In my experience* project. Through these channels, when they enter the web page, students are asked what defines a good student life and encouraged to write their experiences anonymously in the Sensemaker web program. The web page states “**What defines good student lives?** We invite you to describe experiences from your life as a student in Trondheim. Combining your experiences with others will contribute in developing measures made to improve the life quality of all students” [16]. There is a button on the webpage that students can press to share their experience. The descriptions are based on an open-ended question and are fully unaffected by a researcher.

The experience they describe can be related to personal, social, or academic life. It may be positive or negative, a significant or a minor event [16]. It was possible to answer more than once, however, there is not reason to believe that answering more than once was of relevance for the students. After writing the story, the Sensemaker tool asked participants to interpret their own story within preconceived categories. The categories asked for information such as “regarding your story, who did you receive support from, university, parents or friends”. They were also asked to register type of housing, number of years at university, area of education, spare time activities, if this experience is common to them, and how the experience affected them. The categories were constructed by Sensemaker with no influence from the authors and were not a part of the data material for this study. The data material in this study consists of experiences that participants considered to have had a major impact on the participants’ student life.

Before extracting the data relevant to this study, the material consisted of 430 descriptions. After filtering for the inclusion criteria, the data consisted of a total of 12,829 words based on 171 written descriptions. Data were presented to the authors in an Excel file where we were able to extract the descriptions according to the inclusion criteria.

### Analysis

Analysis of the data were facilitated by NVivo, using thematic analysis. Thematic analysis was considered appropriate as it offers a structured method for analysing data, ensuring that the process is both comprehensive and clear. Also in thematic analysis, themes are derived from the data itself rather than being imposed by the researcher. This helps ensure that the findings are closely aligned with participants’ actual experiences and perspectives. The analysis process was performed by the first author, and the results and development of themes was discussed among the authors to find the relevant grouping of themes. Due to the study’s focus on beneficial experiences, descriptions that were categorized as positive, very positive, neutral or both positive and negative were analysed.

Braun and Clarke [14] describe thematic analysis as an accessible and flexible method for qualitative analysis. The method consists of six steps to identify themes in the data, according to Braun and Clarke [14]: (1) Familiarizing yourself with the data; the data were thoroughly read with an inductive approach to obtain an impression of the content, mainly by the first author. The dataset was included in NVivo and read although again while taking notes on possible ideas for coding, such as “housing” or “outdoor activities”. (2) Generating initial codes - features that appeared interesting were identified and organized into groups identified by the first author and discussed by all authors. The dataset was critically assessed to determine if there was a need for additional codes. (3) Searching for themes - the different codes were sorted into themes based on the relevance for the research question. (4) Reviewing themes - the themes were reviewed and revised by the first author and discussed by all authors repeatedly to ensure a coherent pattern. Next, the dataset was read through again to see if the themes created matched the existing themes. Some themes were revised in this process. (5) Defining and naming themes- the themes were analysed to identify which features appeared interesting and relevant, and how they related to the research theme. (6) Final analysis and write-up- a final analytic description was written based on the themes, and data- extracts were chosen to demonstrate the prevalence of the themes. During the analytical process, the research question was printed as a headline in the dataset to ensure searching for the relevant meanings, in addition to repeated discussions by the authors.

According to Braun and Clarke [10], prevalence is one of the factors that decides what qualifies as a theme. A theme can also be constructed if it catches something important about the research question.

## Results

The results encompass two themes that describes experiences in student life that have been beneficial to mental health. The results point towards a process of finding one’s own way in the student role. The first theme is about the feeling of a new life as a student, and the experiences that were most beneficial for mental health in the beginning of student life, such as the introduction week, finding a group to belong to and feeling welcomed. The other is about managing student life, and the experiences students described as beneficial further on in student life, such as a feeling of mastery, finding your true self and support.

### Theme: The feeling of a new life as a student

A new beginning was expressed in various ways. The descriptions may come from students from all years, but refers to experiences made in the beginning of student life.

The introduction weeks at the university were highlighted as beneficial for student life by most of the respondents, although some also expressed scepticism before starting as a student. The introduction week was described as important both for the social environment in class and for more direct personal reasons. It was closely attached to other beneficial experiences, such as finding new friends and finding a group to which they belonged. The role of the mentors (leaders of the introduction week groups) was also highlighted, and an investment from the mentors on how much effort they put into the welcoming process was regarded as crucial. Input from the mentors was described as making the students feel welcomed, enhanced the feeling of belonging to a group, and made the process of having new acquaintances, friendships, and a welcoming social environment in class easier. Some described that the introduction weeks made the start of student life less worrisome.

*The introduction week was a big positive…I met a bunch of new people who later became friends. The term had not started yet*,* so we had no worries*,* kind of (73).*

Feeling welcomed was described as essential for creating a good student life. This feeling could be related to many areas and was articulated by students, mentors (leaders of the introduction week groups), fellow students in the student societies and line associations, and university staff. A feeling that everyone wished them well was described as essential.

*I was very positively surprised to see how well students can welcome each other and that people were genuinely interested in getting to know everyone (410).*

Being included in student society was the experience mentioned most frequently in the data, mostly as an overall positive experience. Negative experiences with the student societies were also described by those who were refused roles within a society; some of them in several student society groups, which encouraged a feeling of rejection. However, all students were over time accepted into a society and described the inclusion and participation as a highly beneficial experience. The dimension of feeling welcomed was essential, in addition to having a feeling of belonging to a group of people with the same interests, goals, and purpose. Dimensions such as friendship and managing new challenges were also strongly related to this experience, and students reported that being included in a student society was experienced as life changing.

*The thing that has meant most for me in my student life is to be involved in a student society. I did not understand how great it was until I became a part of it*,* but today I thank myself every day for doing so. You get a feeling of belonging and being part of a community that is priceless (258).*

Recognizing one’s own identity was also highlighted as beneficial. This was described as a chance to start over, through transitions such as moving away from home or ending a bad relationship, or in other ways changing an unsatisfying situation. It could also be described as merely starting a new life, which gave an opportunity to see oneself in a different light and with different perspectives, and through that, attaining a new view on one’s own identity, preferences, and interests. Even negative experiences that turned positive was described as an opportunity for a change in perspective.

*Me and my partner split up. It gave me the opportunity to redefine who I am and to grow as a person and experience more (364).*

Beneficial experiences with relationships pointed to several kinds of relationships, both romantic and friendships, that had started during introduction weeks, in student societies and in classroom settings. Moving away from home and finding one’s “true self” in new surroundings with new friends and a new way of living were essential in this theme. Establishing new friendships was expressed by some as the main positive experience of becoming a student. This was sometimes a surprisingly positive experience when starting student life. Rewarding and enriching were also terms that were used to describe the meaning of friendship. Some described the dimension of finding someone like yourself, people who were like minded, and through that experiencing a feeling of belonging.

*Something that meant a lot for me as a student was when I finally felt that I had good friends at the university. People from my study program that I was comfortable with and that I could hang out with in my spare time (114).*

Although some of the students were in the 1st or 2nd year of their studies, the friendships were regarded as lifelong. The descriptions stated a clear distinction between acquaintances, of whom there were many, and those who were regarded as true, close friends.

A feeling of mastery and increased self-confidence was also expressed through descriptions on managing new challenges. In the descriptions, this was also related to the development of one’s own identity and finding one’s strength and limitations when faced with new challenges. New acquaintances and environments laid the foundation for novel experiences. According to the descriptions, this allowed the students to grow in novel situations and discover new abilities.

*I got involved with a student society and got the opportunity to challenge myself in a friendly environment where you are allowed to make mistakes (281).*

### Theme: Managing student life

While the descriptions about beginning student life were related to starting a new chapter, the descriptions about ongoing life as a student focused on other experiences. A feeling of acceptance in an environment that was chosen by themselves and not related to family and childhood friends was highlighted. Additionally, safety and belonging to a group, often referred to as family, were crucial to some students’ perception of good mental health. Being your true self was regarded as important, and social activities were considered a significant area for playing out the new role as a student and adult.

Finding your true self was highlighted in several ways and in diverse areas. When no longer being supported by established networks (i.e., parents and childhood friends), students new to university life were required to navigate an independent path, deciding what interests they wanted to pursue and how they wanted to live their life. Finding your true self could also affect the choice of academic programme; one student explained that in a new environment, it became clearer what he or she truly wanted.

*After the first semester in my original program*,* I found out that this was not what I wanted. I had to go through a process where I had to accept that I had to start a new and find a new study program (31).*

Their professional and private interests also became clearer as the influence from family and childhood friends weakened. With an array of options for leisure time pursuits, it also had to be clearer who they felt they truly are and how they want to prioritize their leisure time.

Taking part in social activities was particularly prominent in the descriptions. Although the activities could be reported within the frames of, for example, student society participation or class environment, taking part in social activities and through belonging to a social group was reported as a paramount beneficial experience. Examples of social activities were volleyball, football, cinnamon bun Wednesday or free oatmeal breakfast at the student café. It was expressed through the joy of belonging to a group and doing activities together with someone. The happenings in the student cafes were arranged by the students’ welfare organization and created a meeting point and an arena for socializing.

*On Mondays and Thursdays there’s free oatmeal before 10 am in the students’ café. It gives me a proper boost in both getting to campus and to start the day early. It is so nice when the whole bunch is sitting there with our bowls of oatmeal and a coffee. It’s an everyday joy that builds relationships and means a lot in the long run (179).*

The relationship with university staff and the importance of feeling acknowledged were described by several students. Lecturers showing an active and serious interest in students’ ideas were regarded as creating a basis for growth. It raised self-confidence and helped find the right path. The engagement and enthusiasm from academic staff were described as highly motivating and had a great effect on the academic choices made throughout the course.

*I was met with an interest in my ideas as a student*,* and suggestions on how to develop them…the institute and their enthusiasm for students’ ideas has made my final years as a student incredibly colourful and motivating (100).*

Students also highlighted the importance of having a supportive housing arrangement when living with friends or co- students. A period of trying different kinds of housing was necessary for some of the participants, and through that process, they described feeling more secure in what their own needs were. Some lived with people they knew when relocating to study, others described meeting someone during the first weeks or in student societies that they later became close to and moved in with. Roommates being regarded as a family was highlighted. According to some descriptions, the sense of having a family gave them a feeling of belonging somewhere.

*Living with three of my best friends is my best experience after two years as a student (191).*

Finding a feeling of inner balance was another beneficial experience that was reported in the descriptions. This was illustrated in the descriptions when topics such as meditation, peaceful surroundings, enjoying nature, or architecture were articulated. Finding a balance in everyday life was highlighted as a positive contribution to wellbeing but also as fundamental in some of the descriptions. One of the descriptions argued that:

*To meditate for one hour every day for the last six months has changed my life completely (125).*

The result of this analysis is that the descriptions point in two directions: one is the experience of starting as a student, and the other is the beneficial experiences that follow the students on their maturing into their role as students and adults.

## Discussion

The descriptions of students’ experiences were clearly divided into two distinct themes. One consisted of descriptions about the feeling of a new life as a student, and the second describing experiences with managing student life that were beneficial for mental health. Experiences such as being welcomed, being included, belonging to a social group, finding one’s own identity, maturing and developing were all highlighted.

The results indicate that a feeling of being welcomed is important as a basis for creating a good student life. Moving to a new place, with all the uncertainties and rapid and major changes, can challenge young people who may never have left their family home and secure their surroundings before. According to Knoesen and Noede [18], many students experience psychological distress in this highly developmental process. Feeling welcomed can be achieved in many ways. The introduction week was described by several participants as crucial in getting a feeling of being welcomed into the student society. Vigen (2021) describes the introduction week as the time when students learn how to be a student, and the student community is established. He describes the introduction week as a source of inside information on how to be a student and suggests that the introduction week is closely attached to developing a student identity. However, research suggests that the introduction weeks’ high focus on drinking may be challenging for some [19]. Thus, it may be important to facilitate introduction weeks that encourages participation and contribute to empowerment for everyone.

Our study suggests that being included is important and applies to the social part of being a student, such as in student societies, and that inclusion in a student society may affect the rest of their lives as students. It also applies to the interest shown by academic staff that contributes to the students’ feeling of being a professional in their own field and gives a feeling of being included in an academic community. This can be related to Antonovsky’s [13] concept of meaningfulness, which is the motivational dimension of the sense of coherence, and it refers to problems being seen as challenges rather than burdens. Being included in an academic community, as described by the participants, may enhance a feeling of mastery and a belief that problems are solvable, and a sense of being empowered. It is however important to be aware of the power structures in academia, and how this may be experienced as alienating for some students. A safe and including environment is therefor of importance. A feeling of being welcomed and included that were highlighted in the descriptions may also enhance a feeling of belonging. Sense of belonging is one of the strongest predictors of mental health in students [20]. According to Volstad [21], small class sizes and small student and faculty populations contributed to a feeling of belonging to the university. This is also confirmed by Skoglund et al. [19], who found that a sense of belonging needed to be derived from both students’ own effort and facilitation from the university by creating small groups to lower the threshold for establishing new contacts. This suggests that a sense of belonging to a university community both socially and academically is of high importance.

Belonging to a social group is highlighted in our findings and is related to the ability to manage life as a student. In Antonovsky’s sense of coherence, manageability is a key concept, and refers to the extent to which one feels that they have the necessary resources available to meet life’s demands, either in one’s own hands or accessible from others [13]. Our findings suggests that having a sense of manageability and feeling that life as a student can be managed is important. This may also be related to the empowerment process that is a key concept in health promotion [10], students having a sense of control over their own health. However, the very characteristics of starting student life and entering an unknown setting may be experienced as disempowering, thus facilitating arenas and settings that contribute to mental health promotion through empowerment is important. The dimension of empowerment that relates to participation is also found in our results; student societies and civic engagement are regarded paramount positive experiences for students, suggesting belonging to a network also outside the study program a contribution to empowerment and health promotion. Such student groups or communities may provide the social capital that Putnam [22] describes as the collective resources that are available for the individuals in the community. Being part of such social groups may therefore strengthen students’ ability to manage challenges in student life.

The findings in our study show that discovering one’s own identity and maturing into the adult self could be achieved and experienced in various ways. Being accepted and welcomed into an academic environment was important. Having one’s ideas and thoughts taken seriously linked the students to their role as professionals and an identity of a mature professional within their field of knowledge. This applies to Antonovsky’s concept of comprehensibility and his view that it is not possible to deal efficiently with stressors unless one has a clear view of the character of the situation [23]. This may indicate that the students need a clear understanding of the challenges they meet when starting university and in being a student.

Our findings also point to a process of development and experiences that may have a positive impact on the process of discovering one’s platform and true self in student life, and eventually as an adult. From our results, we find that the students themselves should take initiative and be active in entering the arenas where mental health-promoting activities happen, as they are the experts on their own needs. Entering such arenas may require a sense of empowerment, being in control, but it may also facilitate empowerment, being in an environment that is related to their own needs and identity. Thus, seeing the individual (student) as experts on themselves and their own needs [24], taking active part in forming own activities may contribute to an empowering process. This finding is supported by Skoglund et al. [19], who suggests that students should be proactive and actively participate in arenas that promotes mental health. To be filled with positive emotions, and function well psychologically and socially, is described as flourishing [12], and is a commonly used concept in the research field of students and mental health [18, 21]. Establishing an environment in which students actively participate in shaping the spaces that promotes mental health may, in turn, benefit less empowered students by reducing barriers and creating more welcoming environments where all students can flourish. Additionally, the findings suggests that positive experiences occur in many different arenas in student life. Rebholz [25] concludes that universities need to create a salutogenic environment to facilitate, promote and sustain mental well-being. In our study, the importance of students’ civic engagement is described by our participants within the frames of introduction weeks, student societies, class environment and housing facilities. However, taking part in these activities requires the student to feel empowered to take an active role in shaping their own student life, and may thus not be manageable for all students. Therefore, universities and stakeholders should facilitate and support such activities to lower the threshold for all students to participate. According to Keyes [12], an important dimension of well- being is social acceptance, contribution and coherence, all criteria that are likely to be fulfilled within the setting of various student groups, socially or academically. A strong sense of coherence in students may contribute to the ability to utilize the measures that provide positive experiences that makes students feel empowered and promote good mental health. According to Saheb et al. [26], peer-to-peer-based activities are a highly effective strategy for promoting student well-being. This applies to introduction week, facilitating shared households, student societies and an inclusive academic environment. A holistic approach should therefore be taken in universities and with stakeholders to build an environment that facilitates positive experiences for students in their student life.

This study gives unique insight into the stories told by students as an answer to an open-ended question, and a holistic approach that allows students to describe all aspects of student life that have been beneficial in their student life. The experiences that are shared are to-the-point, focused and answered freely regarding scope and details. Analysing short text, however, removes the opportunity for follow up questions or to ask for elaborations. Anonymous descriptions may have contributed to the participants feeling less limitations on which experiences they described.

APA dictionary of psychology describes volunteer bias and the risk of the participants not being representative of the group that is being investigated (ref). There is a potential difference between those who volunteer to research, and those who do not. In this study, it was not possible to reduce this bias; however, it must be taken into account when evaluating the research.

A limitation of this study is the uneven balance between male and female participants, as more female than male students described their experiences in the Sensemaker tool. Research shows that females are more likely to participate in research when the decision of participation is influenced by social connections [27]. Participants in the *In my experience* project are mainly recruited through social connections, and this may be part of the explanation for the low number of male participants. This may affect the generalizability of the findings. While the findings remain pertinent, their generalizability may be diminished when considering the experiences of male students. There may be an interest to conduct research on the male student population and their needs for mental health promotion measures, as their voices may not have been sufficiently heard in this data material.

## Conclusion

Students’ descriptions of experiences in their student life that are beneficial to mental health are mainly focused around two themes. First, a feeling of being welcomed and included contributes to developing and maturing into one’s adult self. Second, in student life, a sense of belonging is regarded as important by students. This sense can be found in student societies and other areas for civic engagement. It can also be found in being accepted into an academic community or environment. To access and utilize the measures that can contribute to good mental health in student life, students need to be active and take initiative, and the mental health-promoting arenas need to be facilitated in a way that makes it accessible for those with less sense of coherence and less ability to take initiative.

The findings in this study contribute to the body of knowledge on students’ mental health and are useful for both university stakeholders and students. The findings can be used by administrative university staff to inform planning of the structure of the university and study programs. Future research may investigate how stakeholders can share this knowledge with academic staff to ensure that they include students in academic life at the university as coming professionals. Further research may also explore how these results may be useful for Student welfare organizations when planning for social activities, housing arrangements and possibilities for civic engagement for students, and to investigate how students may use the knowledge to focus their effort on gaining experiences that are beneficial to mental health both when starting as a student but also during life as a student.

## Data Availability

No datasets were generated or analysed during the current study.
